# Concentric and Eccentric Pedaling-Type Interval Exercise on a Soft Robot for Stable Coronary Artery Disease Patients: Toward a Personalized Protocol

**DOI:** 10.2196/10970

**Published:** 2019-03-27

**Authors:** Daniel P Fitze, Martino Franchi, Werner L Popp, Severin Ruoss, Silvio Catuogno, Karin Camenisch, Debora Lehmann, Christian M Schmied, David Niederseer, Walter O Frey, Martin Flück

**Affiliations:** 1 Laboratory for Muscle Plasticity Department of Orthopaedics, Balgrist University Hospital University of Zurich Zurich Switzerland; 2 Balgrist Move>Med Swiss Olympic Medical Center Balgrist University Hospital Zurich Switzerland; 3 Spinal Cord Injury Center Balgrist University Hospital Zurich Switzerland; 4 Rehabilitation Engineering Laboratory Department of Health Sciences and Technology ETH Zurich Zurich Switzerland; 5 University Heart Center Zurich, Sports Cardiology Section, Department of Cardiology University Hospital Zurich University of Zurich Zurich Switzerland

**Keywords:** cardiovascular rehabilitation, concentric and eccentric exercise, high-intensity interval training, muscle oxygen saturation, near-infrared spectroscopy, peak oxygen uptake, ramp test, skeletal muscle power, soft robot

## Abstract

**Background:**

Cardiovascular diseases are the leading causes of death worldwide, and coronary artery disease (CAD) is one of the most common causes of death in Europe. Leading cardiac societies recommend exercise as an integral part of cardiovascular rehabilitation because it reduces the morbidity and mortality of patients with CAD. Continuous low-intensity exercise using shortening muscle actions (concentric, CON) is a common training modality during cardiovascular rehabilitation. However, a growing clinical interest has been recently developed in high-intensity interval training (HIIT) for stable patients with CAD. Exercise performed with lengthening muscle actions (eccentric, ECC) could be tolerated better by patients with CAD as they can be performed with higher loads and lower metabolic cost than CON exercise.

**Objective:**

We developed a clinical protocol on a soft robot to compare cardiovascular and muscle effects of repeated and work-matched CON versus ECC pedaling-type interval exercise between patients with CAD during cardiovascular rehabilitation. This study aims to ascertain whether the developed training protocols affect peak oxygen uptake (VO_2peak_), peak aerobic power output (P_peak_), and parameters of muscle oxygen saturation (SmO_2_) during exercise, and anaerobic muscle power.

**Methods:**

We will randomize 20-30 subjects to either the CON or ECC group. Both groups will perform a ramp test to exhaustion before and after the training period to measure cardiovascular parameters and SmO_2_. Moreover, the aerobic skeletal muscle power (P_peak_) is measured weekly during the 8-week training period using a simulated squat jump and a counter movement jump on the soft robot and used to adjust the training load. The pedaling-type interval exercise on the soft robot is performed involving either CON or ECC muscle actions. The soft robotic device being used is a closed kinetic chain, force-controlled interactive training, and testing device for the lower extremities, which consists of two independent pedals and free footplates that are operated by pneumatic artificial muscles.

**Results:**

The first patients with CAD, who completed the training, showed protocol-specific improvements, reflecting, in part, the lower aerobic training status of the patient completing the CON protocol. Rehabilitation under the CON protocol, more than under the ECC protocol, improved cardiovascular parameters, that is, VO_2peak_ (+26% vs −6%), and P_peak_ (+20% vs 0%), and exaggerated muscle deoxygenation during the ramp test (248% vs 49%). Conversely, markers of metabolic stress and recovery from the exhaustive ramp test improved more after the ECC than the CON protocol, that is, peak blood lactate (−9% vs +20%) and peak SmO_2_ (+7% vs −7%). Anaerobic muscle power only improved after the CON protocol (+18% vs −15%).

**Conclusions:**

This study indicates the potential of the implemented CON and ECC protocols of pedaling-type interval exercise to improve oxygen metabolism of exercised muscle groups while maintaining or even increasing the P_peak_. The ECC training protocol seemingly provided a lower cardiovascular stimulus in patients with CAD while specifically enhancing the reoxygenation and blood lactate clearance in recruited muscle groups during recovery from exercise.

**Trial Registration:**

ClinicalTrials.gov NCT02845063; https://clinicaltrials.gov/ct2/show/NCT02845063

## Introduction

Diseases of the cardiovascular system cause >4 million deaths annually in Europe, and coronary artery disease (CAD) is one of the most common causes responsible for approximately 1.8 million deaths [1]. According to the Swiss Federal Statistical Office, diseases of the cardiovascular system are the third most frequent cause of hospitalization and the most frequent cause of death in Switzerland [2,3]. The inflammatory process of atherosclerosis is the main cause of CAD [4]. Progression of the disease increases the risk of angina pectoris, myocardial infarction, and cardiac arrest.

According to the Swiss Heart Foundation, common risk factors for CAD are hypertension, hyperlipidemia, obesity, diabetes mellitus, smoking, and physical inactivity [5]. Various studies have shown that exercise reduces morbidity and mortality of patients with CAD [6-8]. Positive effects of exercise in patients with CAD include improved cardiovascular and muscular function, quality of life, and the reduction of depressive symptoms and psychological stress [9]. The molecular mechanisms on how exercise is beneficial for patients with CAD range from the normalization of endothelial dysfunction to vasculogenesis through endothelial progenitor cells [10]. Exercise is, therefore, recommended by leading cardiac societies as an integral part of cardiovascular rehabilitation [11].

The continuous low-to-moderate-intensity exercise was traditionally the training modality chosen for cardiovascular rehabilitation. Although this training method is considered to be safe and practicable and has almost no contraindications for stable patients with CAD, a growing clinical interest in high-intensity interval training (HIIT) has been recently developed for stable patients with CAD [12]; this type of training is characterized by high-intensity periods of exercise, which are alternated by rest or low-intensity periods [13]. In terms of improving the peak oxygen uptake (VO_2peak_), HIIT has shown to produce superior outcomes compared with continuous low-to-moderate-intensity exercise in patients with CAD [14-16]. VO_2peak_ has shown to be an independent predictor of morbidity and mortality of cardiovascular diseases [17-19]. Therefore, it represents an important index when evaluating adaptations to exercise regimes during cardiovascular rehabilitation.

Often the training is performed on a cycle ergometer; thus, it mainly involves shortening (concentric, CON) muscle actions. However, lengthening (eccentric, ECC) muscle actions may be better tolerated by patients with CAD in clinical settings [20,21] because it can be performed with higher loads and lower metabolic cost than CON exercise [22-24], as shown by studies conducted on ECC ergometers [25-27]. Following such evidence, we have recently compared the cardiovascular and muscular adaptations to work-matched CON and ECC pedaling-type interval exercise of physically active and healthy subjects at an intensity being used during cardiovascular rehabilitation on a soft robot [28]; we found that indices of cardiovascular strain, such as VO_2peak_, peak ventilation, peak cardiac output, and blood lactate (BL) values, were lower during ECC compared with CON exercise.

In this ongoing study, we developed a clinical protocol on a soft robot to compare work-matched CON with ECC pedaling-type interval exercise for patients with CAD during cardiovascular rehabilitation. This study aims to find out whether the developed training protocols affect VO_2peak_, as well as aerobic muscle function (based on peak aerobic power output and muscle oxygen saturation), of patients with CAD during cardiovascular rehabilitation. We hypothesize that the CON and ECC protocol lead to distinct cardiovascular and muscular adaptations. This study presents preliminary data for cardiovascular and muscular adaptations to the CON and ECC exercise protocol in the first 2 patients with CAD.

## Methods

### Recruitment and Ethics

For the ongoing study [29], 10-15 patients for each experimental group are recruited by the Department of Cardiology of the University Hospital Zurich (Zurich, Switzerland). The study is approved by the Ethics Committee of the Canton of Zurich on March 23, 2015 (project number KEK-ZH-No. 2014-0319). All investigations were and will be conducted in accordance with the ethical standards of the Declaration of Helsinki of 1964.

#### Inclusion Criteria

Subjects who meet all the following inclusion criteria may be included in the study: aged between 20 and 70 years; stable coronary heart patients without ischemia; left ventricular ejection fraction >50%; drug therapy with angiotensin-converting enzyme inhibitors; VO_2peak_ of >86% of the medical target value; voluntary participation in the study; and written informed consent.

#### Exclusion Criteria

If one or more of the following exclusion criteria are met, subjects cannot be included in the study: relevant valvular heart disease; arterial hypertension (blood pressure at rest >140/90); arrhythmogenic cardiomyopathy; angiotensin-converting enzyme inhibitor intolerance; contraindication for ethical reasons; known or suspected noncompliance with the study plan; drug or alcohol disease; the inability of a patient to follow the study procedure (eg, owing to language problems, mental illnesses, and dementia); participation in another clinical study within the last 30 days prior inclusion and during the study; and other clinically significant comorbidities (cardiac arrhythmia, renal insufficiency, hepatic dysfunction, connective tissue disease—Marfan syndrome, Ehlers-Danlos syndrome).

### Clinical Background of Patients

The first patient is a 63-year-old male patient with a known coronary 3-vessel disease. Twelve years ago, he underwent a coronary-aortic bypass surgery procedure with 3 grafts in the setting of an acute elevation of the ST-segment of the electrocardiogram, indicating a total occlusion of a coronary artery (ie, ST-elevation myocardial infarction) 1 month earlier. The left ventricular systolic function appeared normal. The last coronary angiography was 3 years ago where 2 drug-eluting stents were deployed. His cardiovascular risk profile comprises arterial hypertension, previous smoking (30 pack-years), hypercholesterinemia, diabetes, and positive family history for a premature cardiovascular disease. The arterial hypertension is currently well treated with combination therapy of perindopril, indapamide, and amlodipine. Diabetes did not necessitate treatment. Hemoglobin A1c, a long-term indicator of the quality of glucose control in diabetes, is currently 7.1%. Furthermore, he is on aspirin, bisoprolol, and rosuvastatin. Currently, he is free of symptoms; however, in light of secondary preventive aims, cardiovascular rehabilitation is warranted.

The second patient is a 62-year-old male who experienced an ST-elevation myocardial infarction 2 years earlier. The left anterior descending artery was successfully treated with 3 drug-eluting stents. On echocardiography, a normal left ventricular ejection fraction was noted; however, with regional wall motion abnormalities corresponding to the territory of the infarction (anterior and anteroseptal). His cardiovascular risk profile comprises a history of smoking (20 pack-years), treated hypercholesteremia, obesity (body mass index, 30 kg/m^2^), and psychosocial stress. His present medication comprises aspirin, bisoprolol, lisinopril, and rosuvastatin.

Both patients are currently free of symptoms; however, in light of secondary preventive aims, cardiovascular rehabilitation in both patients is warranted.

### Study Design

**Figure 1:**
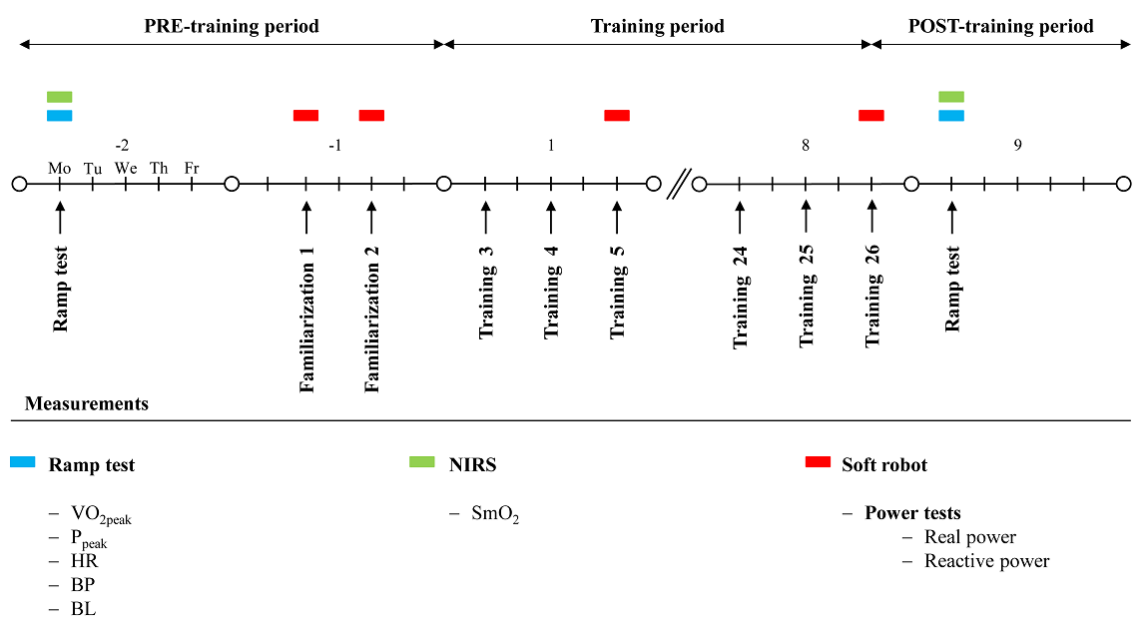
The study design timeline. CON: concentric protocol; ECC: eccentric protocol; PRE: before training period; POST: after training period; VO_2peak_: peak oxygen uptake; P_peak_: peak aerobic power output; HR: heart rate; BP: blood pressure; BL: blood lactate; NIRS: near-infrared spectroscopy; SmO_2_: muscle oxygen saturation.

This study can be divided into 3 periods (Figure 1). The training on the soft robot is performed following either a CON or ECC protocol. Before the training period (PRE) during the first week, subjects complete a ramp test on a cycle ergometer to assess cardiovascular and aerobic muscular parameters. Subjects are then allocated into a CON or ECC group. In the second week, there are 2 training sessions to familiarize subjects with exercise on the soft robot. Before the 2 familiarizations and before every third training session, anaerobic muscle power is monitored using 2 power tests on the soft robot. Throughout the 8-week training period from the third to the tenth week, a pedaling-type interval exercise on the soft robot is completed, involving either CON or ECC muscle actions. The training is performed 3 times a week on nonconsecutive days. The training volume and intensity are progressively increased over the 8-week training period. In addition, the training load, training power, and positive or negative work are measured in all training sessions on the soft robot to monitor the training stimulus. After the training period (POST), subjects repeat the ramp test on the cycle ergometer to acquire POST values.

### Ramp Test

The ramp test is completed in the exercise physiology lab of the Swiss Olympic Medical Center Balgrist Move>Med to determine cardiovascular parameters based on spiroergometry (VO_2peak_, P_peak_, heart rate [HR], and blood pressure) and SmO_2_ based on near-infrared spectroscopy (NIRS). Before the ramp test, anthropometric data (height and body mass) of subjects are measured, and the body mass index is calculated. Subsequently, subjects fill out the Physical Activity Readiness Questionnaire. A resting electrocardiogram measurement is then performed and checked by a physician to ensure that subjects can conduct the ramp test. The exercise electrocardiogram during the ramp test is continuously monitored by the physician.

Subjects perform the test in an upright sitting position in an air-conditioned laboratory on an electrically braked cycle ergometer (ergoselect 200, ergoline). To determine VO_2peak_, pulmonary gas exchange is measured with a spiroergometry measuring system (MetaLyzer 3B-R2, CORTEX Biophysics). The HR is measured continuously using an HR monitor (SUUNTO t6d, SUUNTO). Systolic and diastolic blood pressure (Sys BP and Dia BP, respectively) are measured on the upper right arm every 2 minutes using a BP monitor (Suntec Tango+, Suntec Medical Inc). In addition, BL is measured by collecting a sample of blood from the earlobe every 2 minutes using a lancing device (Akku-Check, Safe-T-Pro-Plus, Roche Diabetes Care) and a BL monitor (Biosen C-Line, EKF-diagnostic). Details on the NIRS measurement during the ramp test are provided in the following section.

The test protocol is modified as described elsewhere [30]. It starts with a 3-minute rest period, where subjects are asked to sit still on the cycle ergometer without pedaling, while maintaining a normal breathing pattern. Subsequently, subjects begin pedaling at an initial power of 25 W. The power is then increased in 5-W increments every 20 seconds (15 W*min^−1^). Subjects are asked to keep a constant self-chosen pedal cadence throughout the test (optimally between 70 and 100 rpm). The test is stopped when subjects experience volitional exhaustion and are not able to maintain the target pedal cadence. After the stop, there is an 8-minute rest period.

### Near-Infrared Spectroscopy

A muscle oxygen monitor (Moxy, Fortiori Design LLC), based on the NIRS technology, is used to measure SmO_2_ during the ramp test noninvasively. The Moxy Monitor uses 4 different light sources covering wavelengths ranging from 630 to 850 nm and a modified Beer-Lambert law to perform measurements of SmO_2_ [31]. SmO_2_ refers to the percentage of hemoglobin and myoglobin that have bound oxygen of the investigated muscle [31].

**Figure 2:**
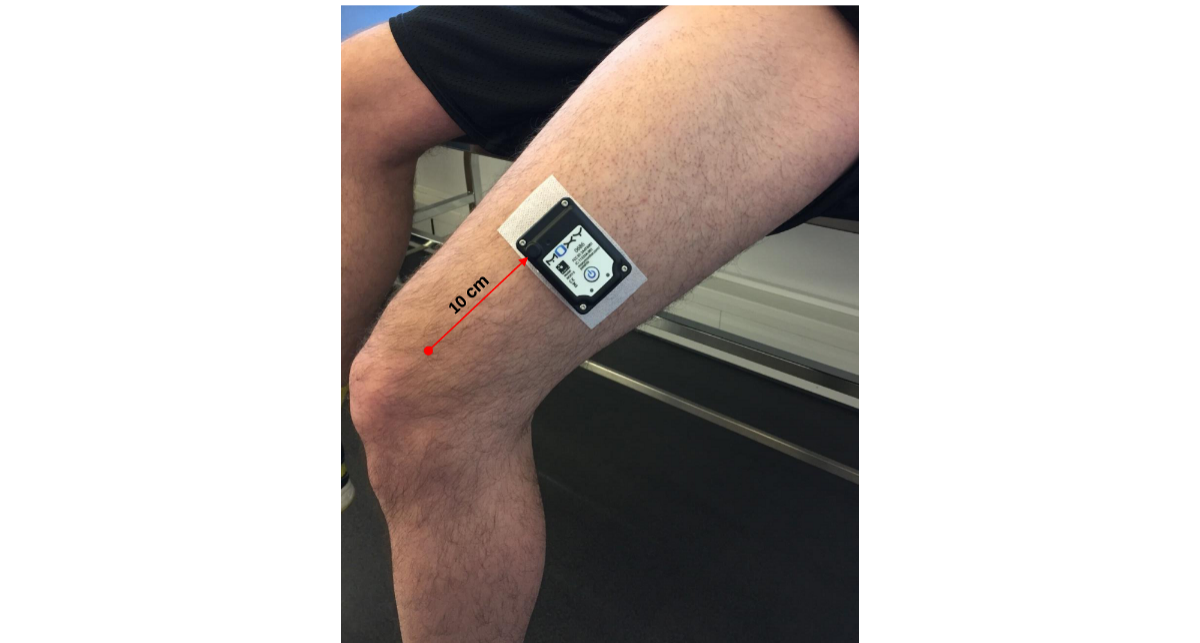
Moxy monitor placement.

The sensor is placed on the lower third of the *m. vastus lateralis* in the middle of the muscle belly on the left leg of subjects (Figure 2). The sensor is placed 10 cm above the upper lateral point of the patella along the axis of the leg. After the placement, the NIRS device is covered with an adhesive nonwoven fabric to protect it from ambient light. Prior to the placement, if necessary, the skin site is shaved using a disposable razor (Gallant, Dynarex) and cleaned with an alcohol swab (Webcol, Covidien). The sensor attachment is carried out using an attachment tape (Moxy Adhesive Attachments, Fortiori Design LLC). To protect the NIRS device from ambient light, it is covered with an adhesive nonwoven fabric (Hypafix, BSN Medical).

### Soft Robot

#### Description of the Soft Robotic Device

A technical description of the soft robotic device (Allegro, Dynamic Devices, Zurich, Switzerland) to be used has been rendered before [28]. In brief, the Allegro soft robot is a closed kinetic chain, force-controlled interactive training, and testing device for the lower extremities. The Allegro device has a leg press layout and consists of two independent pedals and free footplates that are connected to pneumatic artificial muscles through shafts and levers. The seat has an adjustable height and a declinable backrest.

Force application to the pedals is controlled through the supply of pressurized air to the artificial muscles via a software-controlled actuation mechanism for each work cycle. The forces and speeds being produced by the artificial muscles, and those being applied by the user, are controlled during each cycle and displayed via a monitor. This visual feedback allows the user to tune its performance to the target workload through a work cycle. In addition to programmable exercise protocols, the Allegro is equipped with several fundamental test protocols allowing to asses motor accuracy, peak force, positive impulse, negative impulse, net impulse, and power for cyclic movements, reaction time, force control, and force steadiness.

#### Pedaling-Type Interval Exercise

**Figure 3:**
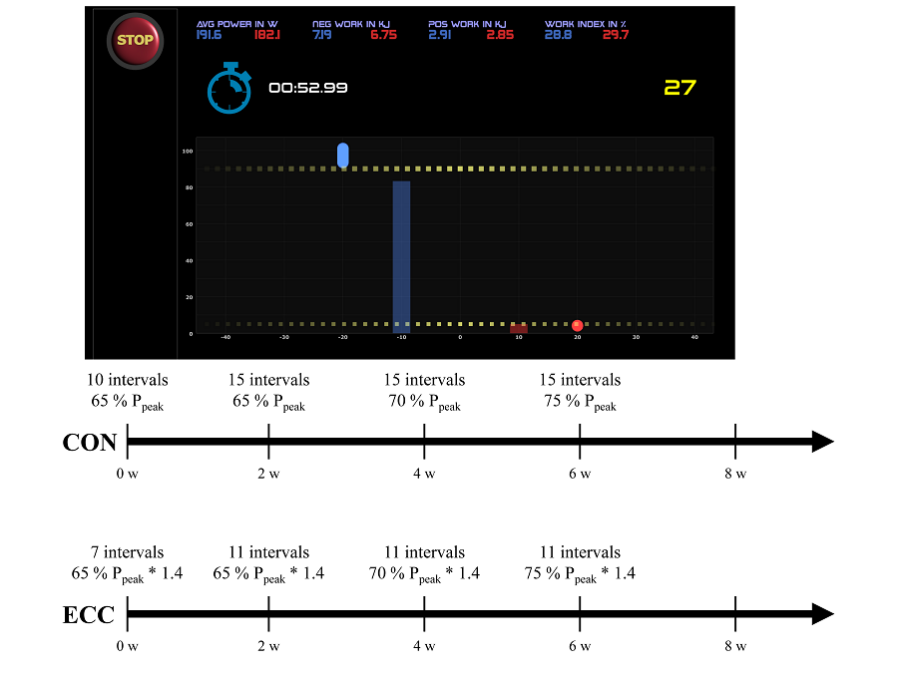
Top: Display of the audiovisual feedback to control training on the soft robot. Bottom: Timeline for the progressive increase of training volume and intensity for the CON and ECC protocol, respectively. ROM: range of motion; CON: concentric protocol; ECC: eccentric protocol; P_peak_: peak power output (Source: Daniel Fitze, Dynamic Devices AG).

The supervised training is carried out at the Swiss Olympic Medical Center Balgrist Move>Med with the *Cardio Power Training* protocol on a soft robot (Allegro, Dynamic Devices). Subjects perform the training 3 times per week with a minimum rest period of 48 h between training sessions. The range of motion (ROM) and movement speed are audiovisually controlled through the integrated screen (Figure 3). Dotted yellow lines, the starting and end knee joint flexion angle and, thus, determine the ROM. During the work phase, the blue and red bar alternately move up and down and, thus, set the movement speed. Blue and red dots represent the current knee joint flexion angle of the left and right leg, respectively. Subjects aim to follow the 2 bars and adhere to the defined ROM. ROM is set to a knee joint flexion angle of 5°-90° and movement speed is 30 rpm. Each interval comprises 1-minute work and 1-minute passive rest period. Training volume and training load are progressively increased over the 8-week training period (Figure 3). For the CON group, the training intensity and volume are determined as follows. During the first 2 weeks, the external load is set to 65% of P_peak_ achieved on the cycle ergometer during the ramp test, and the training comprises 10 consecutive intervals. During the third and fourth week of training, the volume is increased to 15 intervals without changing the external load. During the fifth and sixth week of training, the external load is increased to 70% of P_peak_ while maintaining the volume. During the seventh and eighth week of training, the external load is further increased to 75% of P_peak_. For the ECC group, the calculated training loads are multiplied by factor 1.4. To ensure that both groups perform the same external work, the ECC group completes only 7 and 11 intervals, respectively.

#### Power Tests

A *Real Power* and a *Reactive Power* test are performed on the soft robot to monitor anaerobic muscle power of the lower extremities. The power tests are performed before and after both familiarizations and every third training session.

**Figure 4:**
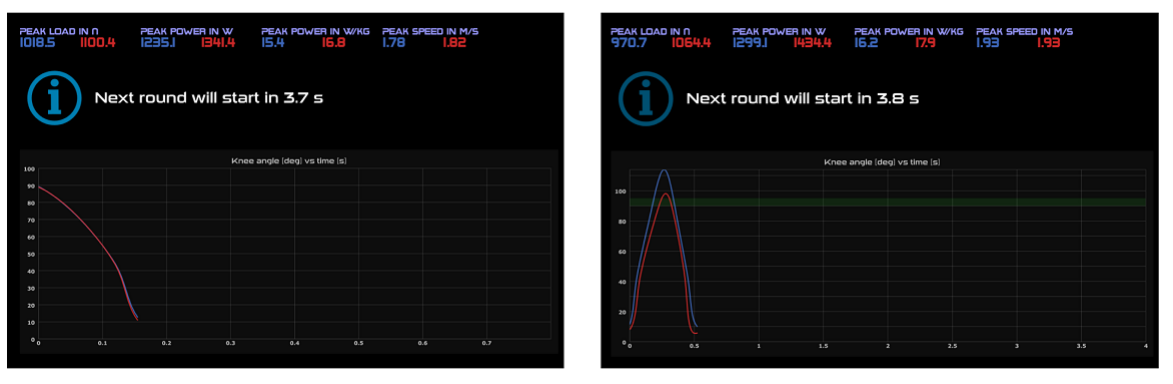
Left: Display of the visual feedback during the Real Power test on the soft robot. Blue and red lines, the knee joint flexion angle of the left and right leg versus time. Right: Display of the visual feedback during the Reactive Power test on the soft robot (source: Daniel Fitze, Dynamic Devices AG).

The *Real Power* test is used to determine anaerobic muscle power during a simulated squat jump. The external load corresponds to 50% of the body mass per leg. Subjects are instructed to flex both legs until a knee joint flexion angle of 90°. With the command “Push,” subjects have to extend both legs as fast as possible. Figure 4 (left) illustrates the provided visual feedback.

The *Reactive Power* test is used to determine the skeletal muscle reactive power during a simulated countermovement jump. The external load corresponds to 50% of the body mass per leg. Subjects have to flex and extend both legs as fast as possible. The test is considered valid if both legs reach a knee joint flexion angle of 90°. Figure 4 (right) illustrates the provided visual feedback.

In Figure 4, the blue and red lines show the knee joint flexion angle of the left and right leg versus time. Green zone, the target knee joint flexion angle of 90°.

A previous characterization has indicated high reliability and comparability for values of force and power between soft robot-based measurements of simulated squat jumps and force-plate measurements during real squat jumps [28].

### Data Analysis

#### Ramp Test

VO_2peak_ is defined as the highest VO_2_ value averaged over a period of 30 s. P_peak_ corresponds to the peak aerobic power output on the cycle ergometer. Peak heart rate (HR_peak_) represents the peak 5-s HR value. Peak systolic and diastolic blood pressure (Sys BP_peak_, Dia BP_peak_, respectively), and peak blood lactate (BL_peak_) define the measured peak values achieved during the ramp test.

#### Near-Infrared Spectroscopy

**Figure 5:**
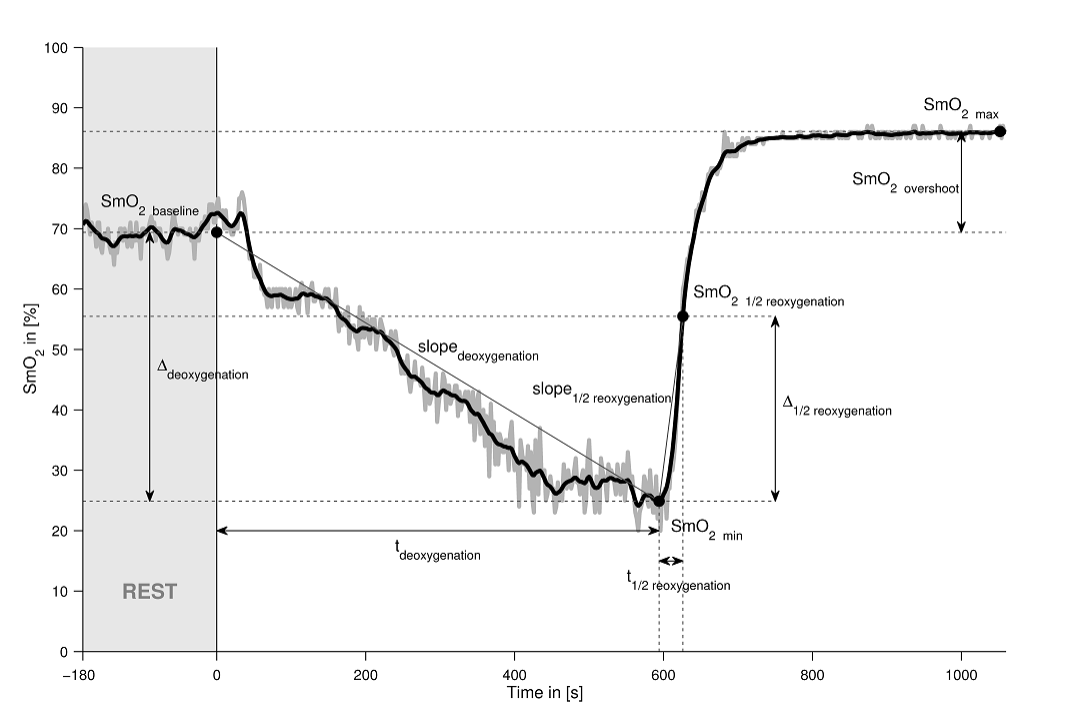
The representative example of the SmO_2_ course during the ramp test of a healthy subject including raw data, processed data, and different parameters. SmO_2_: muscle oxygen saturation; t: time; min: minimum; max: maximum.

Figure 5 shows a representative example of the SmO_2_ course during the ramp test of a healthy subject including raw data, processed data, and different parameters. Data processing and analysis are performed using a data processing program (MATLAB 2015a, The MathWorks). SmO_2_ data are filtered using a second-order zero-phase shift Butterworth low-pass filter with a cut-off frequency of 0.03 Hz. Data extraction is performed based on the description of the NIRS signal interpretation provided elsewhere [32]. SmO_2baseline_ represents the mean value of the 3-minute prerest period. The minimum SmO_2_ value during the ramp test (SmO_2 min_) is extracted by taking the last local minimum of the filtered SmO_2_ prior to reoxygenation. Furthermore, ∆_deoxygenation_ is the difference between SmO_2baseline_ and SmO_2min_; *t*_deoxygenation_ is the time from the beginning of the ramp test until SmO_2min_ is reached; slope_deoxygenation_ is calculated using ∆_deoxygenation_ over *t*_deoxygenation_; SmO_2max_ is defined as the highest value achieved within the 8-minute postrest period; SmO_2 1/2reoxygenation_ is defined as 50% of the difference between SmO_2max_ and SmO_2min_; ∆_1/2reoxygenation_ is the difference between SmO_2 1/2reoxygenation_ and SmO_2min_; *t*_1/2reoxygenation_ is defined as the time between SmO_2min_ and SmO_2 1/2reoxygenation_; slope_1/2reoxygenation_ is calculated using ∆_1/2reoxygenation_ over *t*_1/2reoxygenation_; and SmO_2overshoot_ represents the difference between SmO_2max_ and SmO_2baseline_.

#### Soft Robot

For the *Real Power* and *Reactive Power* test on the soft robot, the average and the peak value of the 4 test attempts are extracted.

#### Statistical Analysis

Data will be analyzed for interaction effects of the exercise type on training-induced alterations using a mixed analysis of variance with repeated measures using statistical software (SPSS Statistics 22; IBM). Effects will be localized posthoc with the least significant difference of Fisher. Furthermore, effect sizes and power will be estimated subsequently with publicly available G*Power software (gpower.hhu.de/).

## Results

### Description of Effect

The results of the first 2 patients who completed the CON and ECC protocol, respectively, are listed below as percentage changes. Multimedia Appendix 1, and 3 present absolute PRE and POST values.

### Ramp Test

**Figure 6:**
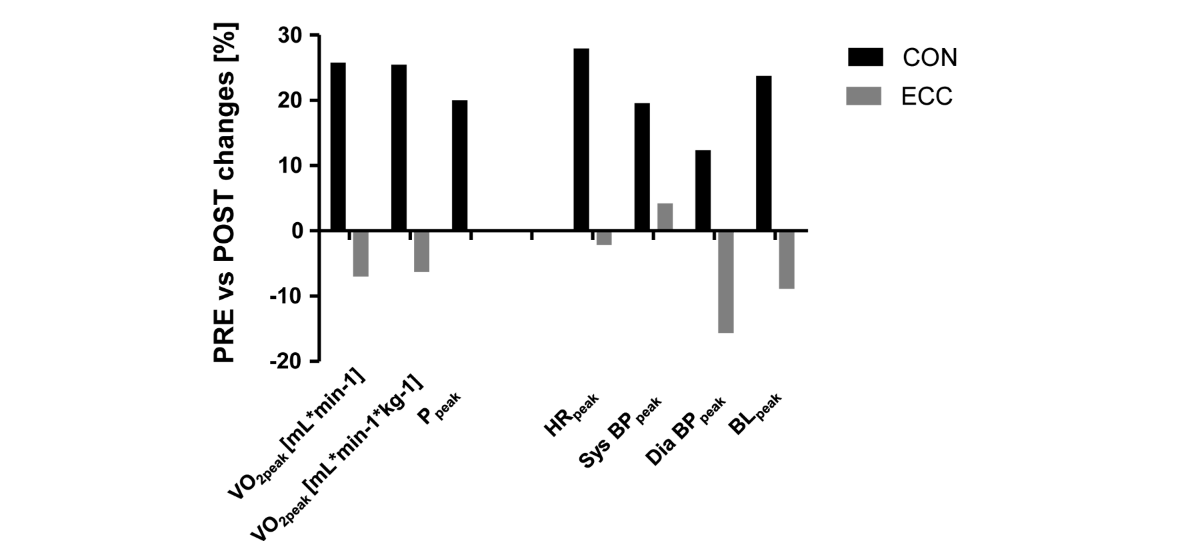
Training-induced changes of cardiorespiratory parameters in the first 2 patients completing the CON or ECC protocols. Bar graph of the percentage changes after versus before the training period (ie, POST vs PRE). VO_2peak_: peak oxygen uptake; P_peak_: peak aerobic power output; HR_peak_: peak heart rate; Sys BP_peak_: peak systolic blood pressure; Dia BP_peak_: peak diastolic blood pressure; BL_peak_: peak blood lactate concentration.

Figure 6 shows training-induced percentage changes in cardiovascular performance. Before training, the patient completing the ECC protocol had better values of cardiovascular performance than the patient completing the CON protocol, that is, absolute VO_2peak_ (3.16 vs 1.94 mL·O_2_·min^−1^), relative VO_2peak_ (29.01 vs 20.9 mL·O_2_·min^−1^·kg^−1^), and P_peak_ (250 vs 150 W). After training, the patient completing the CON protocol demonstrated an increased absolute VO_2peak_ (+26%), relative VO_2peak_ (+26%), and P_peak_ (+20%), when the ECC patient showed reduced values for absolute VO_2peak_ (−7%) and relative VO_2peak_ (−6%), while maintaining P_peak_. HR_peak_ and BL_peak_ concentration during exercise were increased by 28% and 24% after the CON protocol and 2% and 9% reduced after the ECC protocol, respectively.

### Near-Infrared Spectroscopy

**Figure 7:**
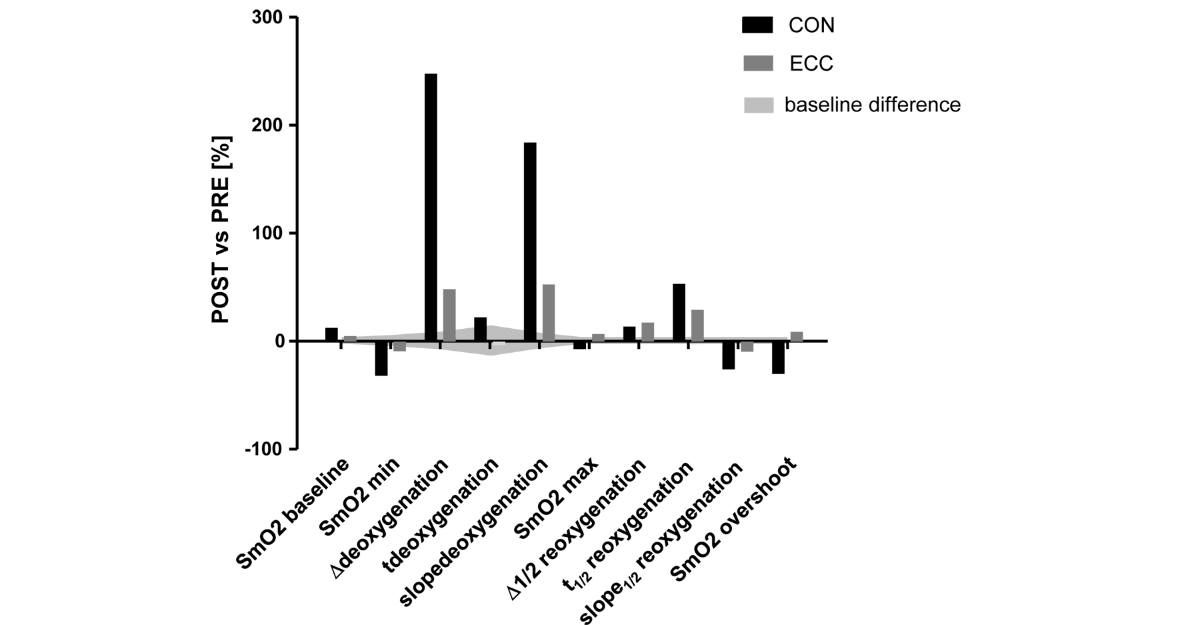
Training-induced changes of SmO_2_ parameters in the first 2 patients completing the respective CON or ECC protocols. Bar graph of the percentage changes POST versus PRE. SmO_2_: muscle oxygen saturation; t: time; min: minimum; max: maximum.

Figure 7 shows training-induced percentage changes of SmO_2_ parameters. Details of the extracted SmO_2_ parameters can be found in the section data analysis. Gray shaded area refers to the observed baseline differences of the measurements prior to training. The minima of SmO_2_ during the ramp test were further reduced by training under either protocol, whereby changes being reflective of muscle deoxygenation were more increased after training for the CON respective to the ECC protocol, that is, SmO_2min_, ∆_deoxygenation_, *t*_deoxygenation_, and slope_deoxygenation_. In contrast, SmO_2max_ and SmO_2overshoot_ increased in the patient completing the ECC protocol, while the values for these parameters decreased in the patient completing the CON protocol (Multimedia Appendix 2).

Before training, the patient entering the ECC protocol had 25% and 23% higher peak real power values of the left and right leg, respectively, than the patient entering the CON protocol (Multimedia Appendix 3). In contrast, the peak power of the left and right leg in the reactive power test was 28% and 19%, respectively, lower for the patient entering the ECC protocol than the patient entering the CON protocol.

**Figure 8:**
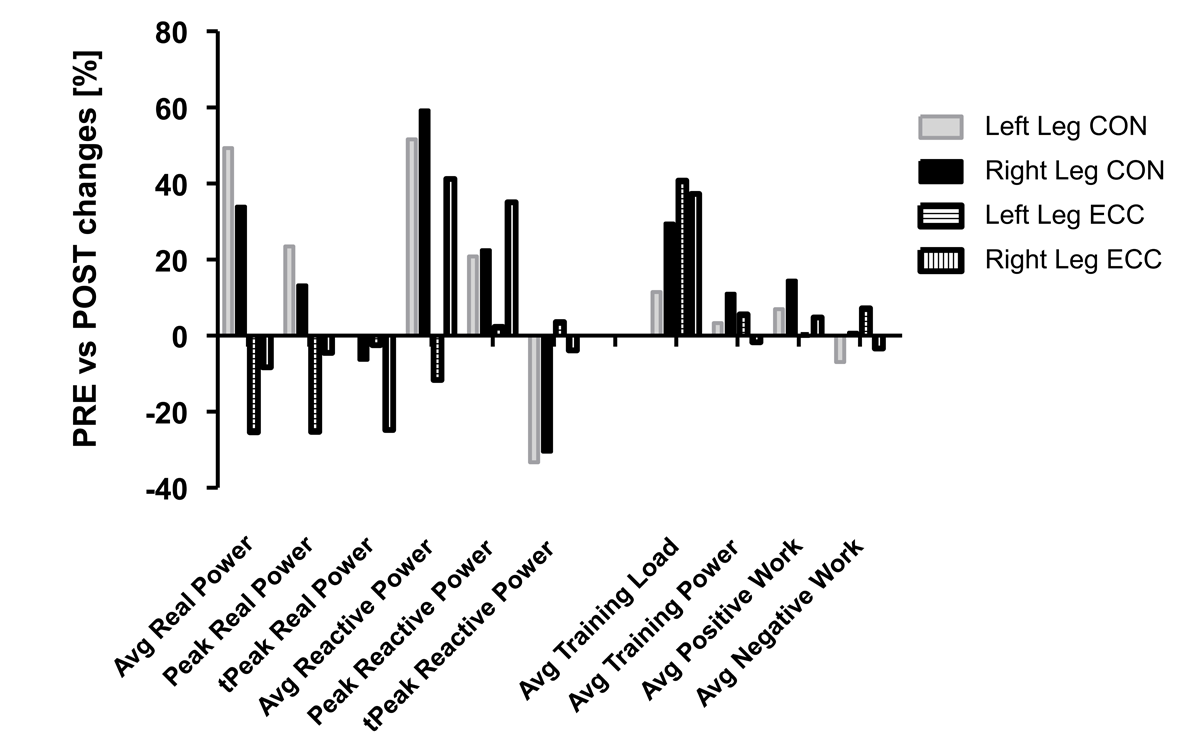
Training-induced changes of the muscle performance on the soft robot for patients completing the respective CON or ECC protocols. Bar graph of the percentage changes POST (after the training period) versus PRE (before the training period). CON: concentric protocol; ECC: eccentric protocol; Avg: average; t_peak_: time to peak.

Figure 8 shows training-induced percentage changes of anaerobic muscle function as assessed separately for both legs on the soft robot. The CON protocol exhibited an improvement in the average power (+49%, +34%) and peak power (+24%, +13%) for the left and right leg during the real power test, when these values were reduced after the ECC protocol (left: −25%, −25%; right: −8%, −5%), despite a larger average increase in the training load. Power during the reactive power test was improved with both protocols, whereby leg differences revealed for the ECC protocol.

## Discussion

### Principal Findings

We developed a novel exercise protocol on an interactive soft robot to investigate the effects of work-matched CON versus ECC pedaling-type interval exercise on cardiovascular and muscular parameters of stable patients with CAD during cardiovascular rehabilitation.

One of the strengths of the protocol is that the measured cardiovascular and muscular parameters have well-documented prognostic relevance in patients with CAD. Furthermore, the combination of HIIT and ECC muscle actions in stable patients with CAD during cardiovascular rehabilitation can be considered innovative. Implementing the combination of these training modalities has the potential to make the exercise protocol practicable and more time-efficient for CAD patients with CAD. A further strength is its personalized approach. The initial power on the training device is determined based on P_peak_ during the ramp test; this allows an individualized control of the training intensity but could also represent a limitation of the study, as the power on the cycle ergometer does not match exactly the power of the soft robot.

### Ramp Test

The data demonstrate that the CON pedaling type of interval exercise protocol is capable of producing a substantial increase in VO_2peak_ in absolute and relative terms (ie, +26%) and P_peak_ (ie, +20%) in patients with CAD. In addition, increased values for HR_peak_, Sys BP_peak_, Dia BP_peak_, and BL_peak_ were found during the ramp test after the training period under the CON protocol. As VO_2peak_ seems to reflect a continuum between health, cardiovascular disease, and death, it is important to design effective programs for exercise-induced gains of VO_2peak_ [14]. The increased VO_2peak_ can lead to a more active lifestyle, which can favor additional preventive benefits; this is assisted by the improved P_peak_, which is related to improved mobility [33].

Previous studies have shown that HIIT is an effective method for improving VO_2peak_ in patients with cardiovascular diseases. A study investigated the effects of HIIT compared with continuous moderate-intensity exercise in stable patients with CAD, showing an increase of the VO_2peak_ by 17.9% in the HIIT group [14]. The exercise protocol consisted of 4-minute intervals at 80%-90% of VO_2peak_ with 3-minute active recovery periods at 50%-60% of VO_2peak_. The increase in VO_2peak_ was significantly higher in the HIIT group compared with the continuous moderate-intensity exercise group (17.9% vs 7.9%). Similar or even better VO_2peak_ improvements were shown in studies that used shorter but more intensive intervals in stable patients with CAD. A 16-week interval training, in which the intervals consisted of 2-minute work phases at 85%-95% of the HR and VO_2_ reserve and 2-minute rest periods at 35%-45% of the HR and VO_2_ reserve, led to a 15% VO_2peak_ improvement in highly functional CAD patients [34]. In addition, a 12-week interval training period, in which subjects completed ten 1-minute phases at 89% separated by 1-minute phases at 10% of peak power output, led to an improvement of the VO_2peak_ of 20% [35].

The aforementioned studies underlined the importance of the training intensity to effectively increase VO_2peak_. HIIT allows patients with CAD to train for longer periods of time at a higher-intensity, as it would be possible with continuous training [36]. However, protocols that use very short intervals, such as Wingate-based HIIT, may not be safe and tolerable for patients with metabolic diseases [12]. The selection of the exercise intensity, the duration of the intervals, and the use of active or passive rest have a profound influence on the acute physiological responses and exercise tolerance in patients with CAD [36]. Therefore, we decided on a protocol consisting of 1-minute work phases and 1-minute phases of passive rest; the results show that a considerable increase in VO_2peak_ can be achieved with the selected training intensity and volume and their progressive increase during the 8-week CON pedaling-type interval exercise protocol on the soft robot. Furthermore, the increased values for HR_peak_, Sys BP_peak_, Dia BP_peak_, and BL_peak_ during the POST ramp test show that the patient was able to exercise to a higher degree.

### Near-Infrared Spectroscopy

A number of changes in SmO_2_ parameters during the ramp test were identified after the training period. The most noteworthy are the changes in the parameters ∆_deoxygenation_ and slope_deoxygenation_, both of which have been more than doubled after training. The increased deoxygenation could indicate a better O_2_ extraction of the *m. vastus lateralis* during the ramp test. As the deoxygenation depends primarily on the O_2_ uptake of the mitochondria [37], this result could reflect the typically observed increased mitochondrial volume density within the examined muscle after the training period.

Exercise-induced changes in the skeletal muscle deoxygenation have been shown in patient populations. In postmyocardial infarction patients, it has been shown that the skeletal muscle deoxygenation was impaired [38] and that a 12-week training period led to increased deoxygenation of the *m. vastus lateralis* during the ramp test [39]. The NIRS measurement performed in addition to the determination of VO_2peak_ can provide information about the peripheral adaptations in the skeletal muscle. As VO_2peak_ is dependent on a central (ie, cardiac output), as well as a peripheral (ie, arteriovenous O_2_ difference), component, and peripheral metabolic adaptations, such as mitochondrial enzyme activity, in the skeletal muscle are critical for improving endurance performance [40], we decided to measure VO_2peak_ and skeletal muscle oxygenation. Assessing skeletal muscle deoxygenation responses can be helpful to clarify peripheral impairment and its relation to reduced VO_2peak_ in patients after myocardial infarction [38].

### Power Tests

An increase in the average and peak power during both soft robot power tests was observed after the training period. Increasing the skeletal muscle power should be another target during cardiovascular rehabilitation because it has been shown to decline earlier and more rapidly than muscle force, and, therefore, it represents a more discriminant predictor of functional performance in older adults [41]. The observed increase can improve the ability of patients to cope with daily activities and, thus, remain active and independent. The former possibility is indicated for the CON protocol by the enhanced real power and reactive power.

### Protocol

The pilot evidence from the first patients with CAD show differing adjustments in cardiovascular performance (and muscle reoxygenation) in response to the ECC and CON protocol. Specifically, we identify that P_peak_ (VO_2peak_) was maintained in the patient after training with the ECC protocol, despite a moderately reduced cardiovascular performance. Hence, it is relevant to consider that the ECC patient demonstrated a better endurance training status than the CON patient before the training. Hence, a considerable part of improvements in the patient exercising on the CON protocol was related to an improvement in the cardiac function (Figure 6). In addition, it is known that at a similar mechanical power, ECC muscle work induces lower metabolic and cardiovascular responses than CON muscle work [42]. Possibly, the effects in the first 2 of our patients reflect interactions between the training state of the patients, and task-specific adaptations to the training that was matched by the mechanical output (ie, work). For the CAD patient training under the ECC protocol, the observations indicate that an increase in the endurance component, for instance, by increasing the number of work intervals, would have been beneficial to improve cardiovascular parameters.

Furthermore, we identified that peak real power was reduced with the ECC protocol and that reactive power was only improved for the right leg while both parameters were improved with the CON protocol. These observations are of interest with respect to the training status prior to entering the protocols and higher training-related increases in the average training load, and the lower metabolic stress and improved muscle reoxygenation with exhaustive exercise in the ramp test, for the ECC compared with the CON protocol. It indicates that the selected ECC exercise protocol affected the muscle strength primarily by affecting bioenergetic pathways and that this is also affected by the initial training status. For the specific ECC patient, a future closer supervision and coaching of how the task was performed with either leg would have been an option to avoid leg differences in adaptation.

Interestingly, the results show a selective improvement in the maximal oxygenation of the knee extensor muscle after recovery from the exhaustive ramp test after training under the ECC protocol; this finding can be related to the reportedly larger gains in capillary perfusion (ie, based on the capillary-to-fiber ratio) after the eccentric type of cycle endurance training [43]. It indicates that the ECC protocol may specifically enhance the rate of muscle oxygenation during recovery from exercise (reviewed in [44]).

### Conclusions

To summarize, this study indicates the potential of the CON pedaling-type interval exercise protocol to increase VO_2peak_, peak aerobic power output, parameters of SmO_2_, and anaerobic muscle power of a patient with CAD during cardiovascular rehabilitation. In addition, our first observations show that the ECC exercise protocol is well tolerated and maintains the peak aerobic peak aerobic power output in relation to lowered metabolic stress and improved oxygen delivery to the recruited muscle group during recovery from exhaustive exercise. The results of the ongoing study, specifically because they allow addressing the performance of each leg individually through the haptic feedback of the soft robot, may contribute to optimizing exercise protocols during cardiovascular rehabilitation.

## Appendix

Multimedia Appendix 1 Cardiorespiratory parameters pre- and posttraining for the patient, which followed the CON and ECC protocol, respectively.

Multimedia Appendix 2 Parameters of muscle oxygen saturation (SmO_2_) pre- and posttraining for the patient, which followed the CON and ECC protocol, respectively.

Multimedia Appendix 3 Parameters of muscle performance pre- and posttraining for the patient, which followed the CON and ECC protocol, respectively.

## Abbreviations

BL_peak_: peak blood lactate
BL: blood lactate
CAD: coronary artery disease
CON: concentric
CAD: coronary artery disease
Dia BP_peak_: peak diastolic blood pressure
ECC: eccentric
HIIT: high-intensity interval training
HR_peak_: peak heart rate
NIRS: near-infrared spectroscopy
POST: after the training period
P_peak_: peak aerobic power output
PRE: before the training period
ROM: range of motion
SmO_2_: muscle oxygen saturation
VO_2peak_: peak oxygen uptake
