# Supplementary material for: Concentric and Eccentric Pedaling-Type Interval Exercise on a Soft Robot for Stable Coronary Artery Disease Patients: Toward a Personalized Protocol
Source: JMIR Res Protoc. 2019 Mar 27;8(3):e10970. doi: 10.2196/10970 (PMC6456820; doi:10.2196/10970)
Supplement: Multimedia Appendix 1 [file resprot_v8i3e10970_app1.pdf]

Table 1: *Cardiorespiratory parameters pre and post training for the patient, which followed the CON and ECC protocol, respectively.*

	VO2peak [mL*min-1]	VO2peak [mL*min-1*min-1]	Ppeak [W]	HRpeak [min-1]	Sys BPpeak [mmHg]	Dia BPpeak [mmHg]	BLpeak [mM]
CON pre	1.94	20.89	150.00	111.00	179.00	89.00	8.22
CON post	2.42	26.03	180.00	142.00	214.00	100.00	10.17
ECC pre	3.16	29.06	250.00	139.00	216.00	96.00	10.83
ECC post	2.94	27.22	250.00	136.00	225.00	81.00	9.87
