# Supplementary material for: Concentric and Eccentric Pedaling-Type Interval Exercise on a Soft Robot for Stable Coronary Artery Disease Patients: Toward a Personalized Protocol
Source: JMIR Res Protoc. 2019 Mar 27;8(3):e10970. doi: 10.2196/10970 (PMC6456820; doi:10.2196/10970)
Supplement: Multimedia Appendix 2 [file resprot_v8i3e10970_app2.pdf]

Table 2: *Parameters of muscle oxygen saturation (SmO2) pre and post training for the patient, which followed the CON and ECC protocol, respectively.*

	SmO2 baseline	SmO2 min	delta deoxygenation	t deoxygenation	slope deoxygenation	SmO2 max	delta1/2 reoxygenation	SmO2 overshoot	t1/2 reoxygenation	slope1/2 reoxygenation
CON pre	43.33	36.45	-6.88	500.00	-0.01	80.00	43.55	36.67	30.00	0.73
CON post	48.91	24.97	-23.94	612.00	-0.04	74.61	49.64	25.70	46.00	0.54
ECC pre	39.16	29.67	-9.49	914.00	-0.01	74.39	44.72	35.23	34.00	0.66
ECC post	41.15	27.05	-14.10	888.00	-0.02	79.62	52.57	38.47	44.00	0.60
