# Supplementary material for: Concentric and Eccentric Pedaling-Type Interval Exercise on a Soft Robot for Stable Coronary Artery Disease Patients: Toward a Personalized Protocol
Source: JMIR Res Protoc. 2019 Mar 27;8(3):e10970. doi: 10.2196/10970 (PMC6456820; doi:10.2196/10970)
Supplement: Multimedia Appendix 3 [file resprot_v8i3e10970_app3.pdf]

Table 3: *Parameters of muscle performance pre and post training for the patient, which followed the CON and ECC protocol, respectively.*

ECC	Left Leg PRE	Left Leg POST	Right Leg PRE	Right Leg POST
**Real Power**				
Average [W]	846.79	631.88	911.99	836.40
PEAK [W]	1204.45	899.92	1317.81	1257.36
tPEAK [sec]	0.15	0.14	0.20	0.15
**Reactive Power**				
Average [W]	172.31	152.19	178.47	251.91
PEAK [W]	952.04	973.89	1047.57	1415.40
tPEAK [sec]	0.42	0.43	0.53	0.51
**Training Load**				
Average [kg]	43.30	60.93	43.66	59.93
**Training Power**				
Average [W]	171.09	180.63	166.77	163.88
**Positive Work**				
Average [W]	2.27	2.27	2.13	2.23
**Negative Work**				
Average [W]	7.95	8.53	7.83	7.57

CON	Left Leg PRE	Left Leg POST	Right Leg PRE	Right Leg POST
**Real Power**				
Average [W]	780.33	1165.33	864.93	1158.30
PEAK [W]	964.00	1190.20	1072.70	1214.20
tPEAK [sec]	0.13	0.13	0.16	0.15
**Reactive Power**				
Average [W]	998.65	1513.48	968.75	1542.33
PEAK [W]	1327.10	1604.10	1286.60	1575.30
tPEAK [sec]	0.75	0.50	0.76	0.53
**Training Load**				
Average [kg]	42.07	46.89	37.09	48.01
**Training Power**				
Average [W]	104.07	107.52	101.50	112.66
**Positive Work**				
Average [W]	4.78	5.11	4.72	5.41
**Negative Work**				
Average [W]	1.45	1.35	1.31	1.32
